# Peak week recommendations for bodybuilders: an evidence based approach

**DOI:** 10.1186/s13102-021-00296-y

**Published:** 2021-06-13

**Authors:** Guillermo Escalante, Scott W. Stevenson, Christopher Barakat, Alan A. Aragon, Brad J. Schoenfeld

**Affiliations:** 1 Department of Kinesiology, California State University- San Bernardino, CA San Bernardino, USA; 2 Integrative Bodybuilding LLC, FL Tampa, USA; 3 Competitive Breed LLC, FL Tampa, USA; 4 Human Performance Laboratory, The University of Tampa, FL Tampa, USA; 5 Department of Family and Consumer Sciences, California State University- Northridge, Los Angeles, CA USA; 6 Health Sciences Department, Lehman College, NY Bronx, USA

**Keywords:** Bodybuilding, Competition, Contest, Peaking

## Abstract

Bodybuilding is a competitive endeavor where a combination of muscle size, symmetry, “conditioning” (low body fat levels), and stage presentation are judged. Success in bodybuilding requires that competitors achieve their peak physique during the day of competition. To this end, competitors have been reported to employ various peaking interventions during the final days leading to competition. Commonly reported peaking strategies include altering exercise and nutritional regimens, including manipulation of macronutrient, water, and electrolyte intake, as well as consumption of various dietary supplements. The primary goals for these interventions are to maximize muscle glycogen content, minimize subcutaneous water, and reduce the risk abdominal bloating to bring about a more aesthetically pleasing physique. Unfortunately, there is a dearth of evidence to support the commonly reported practices employed by bodybuilders during peak week. Hence, the purpose of this article is to critically review the current literature as to the scientific support for pre-contest peaking protocols most commonly employed by bodybuilders and provide evidence-based recommendations as safe and effective strategies on the topic.

## Background

Bodybuilding is a competitive endeavor where a combination of muscle size, symmetry, “conditioning” (low body fat levels), and stage presentation are judged. To be successful, competitors must present their best physique during the day (or days) of the competition. Bodybuilders typically employ periods of 8–22 + weeks of preparation where diet and exercise programs are modified from the off-season in an effort to lose body fat and gain or maintain skeletal muscle mass [1–10]. In the final days of preparation, competitors have been reported to implement interventions to “peak” their body in an effort to maximize contest day aesthetics [11–14]. The interventions often used include altering their exercise regimens as well as their macronutrient, water, and electrolyte intake with the goals of: (1) maximizing muscle glycogen content as a means to enhance muscle “fullness” (i.e. volume), (2) minimizing subcutaneous water (in an effort to look “dry” as opposed to “watery,” thus enhancing muscularity), and (3) minimizing abdominal bloating to maintain a smaller waistline and optimize physique proportion and overall aesthetics [11, 12, 14–17]. While competitors may use natural methods to achieve these goals, self-prescription of potentially hazardous drugs such as insulin and diuretics have been widely reported [8, 18–21].

An observational study gathered information on nutritional peak week and competition day strategies among 81 natural bodybuilders (Males = 59, Females = 22) via a 34-item questionnaire; the survey listed commonly utilized peaking strategies and provided additional space for qualitative information [11]. The vast majority of the participants (93.8 %) reported employing a peaking strategy the week prior to the competition (referred to as “peak week”), with the manipulation of carbohydrate (CHO), water, and/or sodium most commonly reported [11]. The primary stated goal of CHO manipulation was to maximize muscle glycogen concentrations by utilizing principles similar to classical CHO loading [11]. Additionally, competitors manipulated water and/or sodium intake in an effort to induce a diuretic/polyuria effect to flush out superfluous water [11].

In another study, researchers conducted in-depth interviews to identify and describe different dietary strategies used by seven natural male bodybuilders during the off-season, in-season, peak week, and postseason [14]. During peak week, six participants reported using a modified carbohydrate loading regimen to attempt to increase glycogen content. Furthermore, all participants reported manipulating water intake while three simultaneously manipulated sodium intake in an attempt to reduce body water in hopes of creating a leaner look [14].

Although many peak week protocols exist that attempt to enhance aesthetics, research is lacking in regards to the efficacy and safety of the methods commonly used by bodybuilders. Among enhanced bodybuilders, the self-prescription of drugs such as insulin and diuretics have been reported with potentially dangerous outcomes [18–21]. The purpose of this article is to: (1) review the current literature as to the peaking protocols most commonly employed by bodybuilders; (2) provide evidence-based recommendations as to pre-contest peaking strategies for competitors and coaches.

## Main Text

### Carbohydrate Manipulation

Manipulation of carbohydrate intake is a popular pre-contest peaking strategy among bodybuilders [11, 12, 14]. The strategy, generally employed during the week prior to competition, involves substantially limiting carbohydrate intake for several days (often referred to as depletion phase) followed by a brief period of high-carbohydrate consumption, with the goal of achieving a supercompensation of glycogen levels when carbohydrate is “loaded” [22]. Resting muscle glycogen levels with a mixed (normal) diet are ~ 130mmol/kg muscle (wet weight) in trained individuals (a bit higher than sedentary subjects) [23], or roughly 23 g of glycogen (glucosyl units) per kilogram of muscle tissue. Muscle glycogen is organized in the cell in subcellular fractions [24] and stored as a glycogen-glycogenin complex (“granule”) [25] which creates an osmotic effect of pulling water into the cell as glycogen is stored [26, 27], thereby increasing muscle cell volume. Early research suggested that each gram of muscle glycogen stored is accompanied by approximately 3–4 g of intracellular water [28]. This is higher than the commonly referenced value of 2.7 g of water per gram of glycogen, sometimes rounded to 3 g of water per gram of glycogen, which is derived from studies of rat liver [29, 30]. However, the resultant muscle glycogen levels after glycogen loading is highly variable [31], perhaps due to the complexity underlying intramuscular glycogen storage [25]. Similarly, while it is clear that glycogen loading can increase intracellular water content [31], muscle thickness [15], and lean body mass (LBM) estimates [32], the relative extent of intracellular hydration in grams of water per gram of glycogen may vary so greatly that it is not statistically correlated with glycogen content [30].

Although controlled research on the topic is limited for what is optimal for bodybuilders, current evidence does appear to indicate a potential benefit of carbohydrate manipulation as a peaking strategy. A case series by Bamman et al. comprising six male bodybuilders provided initial support of a beneficial effect [1]. The bodybuilders reportedly engaged in a carbohydrate-loading protocol three days prior to competing (mean intake of ~ 290 g/day). Ultrasound measurements taken 24–48 h into this carbohydrate loading period showed a 4.9 % increase in biceps brachii muscle thickness when compared to measures obtained six weeks earlier. While these findings seem to suggest that the carbohydrate loading protocol was effective in acutely enhancing muscle size, it should be noted that the long gap between testing sessions makes it impossible to draw inferences as to causality in this regard. Moreover, the authors of the study did not assess carbohydrate intake during the carbohydrate depletion phase, further clouding the direct effects of the loading protocol. Thus, while results are intriguing, the level of supporting evidence can be considered low.

A recent quasi-experimental trial by de Moraes et al. [15] sheds more objective light on the topic. Twenty-four high-level amateur male bodybuilders were stratified based on whether or not they manipulated carbohydrate as a peaking strategy; the group that manipulated carbohydrate employed a three day depletion phase (leading immediately up to the weigh-in day) followed by a 24-hour loading phase (leading up to competition day). Muscle thickness was measured both at weigh-in and the day of competition. In addition, photos of the competitors taken at these time points were shown to a group of federated bodybuilding judges, who subjectively assessed their physiques; of note, judges were blinded to the competitors’ nutritional practices. Results showed a 3 % increase in muscle size of the upper arms for those who manipulated carb intake prior to competition versus no change in those who did not. Moreover, only the group that manipulated carbohydrate intake showed improvements in subjective aesthetic measures as determined by visual inspection of photos. A potential limitation of the study is that subjects were not drug-tested prior to competition; thus, it remains unknown as to whether the use of anabolic steroids and/or other synthetic substances (i.e., synthol) may have influenced results. Future studies should ascertain via self-report, polygraph and/or blood testing the drug-free/enhanced status of subjects and exclude or compare results based on subject steroid use as well as use of other drugs that may influence water balance (e.g., caffeine - see below).

Recently, members from our group (Schoenfeld and Escalante) carried out a case study where we followed a high-level natural bodybuilder over the course of his contest preparation [33]. Beginning 1-week prior to the date of competition, the competitor markedly reduced carbohydrate intake to < 50 g/day for 3 days (Sunday, Monday, Tuesday) and then loaded carbohydrate to > 450 g/day over the ensuing 2-day period (Wednesday and Thursday). Similar to previous research, ultrasound assessment showed that the peaking strategy acutely increased muscle thickness. In this particular case study, increases were 5 % in the upper extremities and ~ 2 % in the lower extremities; due to the limited available evidence, it is difficult to provide a rationale as to why there was a difference between the muscle groups. Given the subjective findings reported by de Moraes et al. [15], it can be inferred that these results likely were practically meaningful from a competition standpoint.

When considering the totality of current research, evidence suggests that carbohydrate manipulation is a viable peaking strategy to enhance muscle size on contest day; however, the evidence should be considered preliminary given the relative dearth of published studies on the topic. Moreover, the strategy may bring about an increase in gastrointestinal symptoms such as abdominal pain, heartburn, constipation, and diarrhea [15], which in turn may negatively affect the ability to perform optimally on contest day. Thus, competitors should experiment with the strategy at least 2–4 weeks in advance to determine its effects on an individual level and make relevant adjustments as needed.

### Water and Sodium Manipulation

Water and sodium are frequently manipulated by bodybuilders, either independently or concurrently, employing a variety of strategies involving “loading” and restricting both [11], with the goal of minimizing subcutaneous water to maximize the underlying skeletal muscle definition [8, 11, 12, 14, 19, 20]. Bodybuilders have been reported to self-prescribe potentially dangerous pharmaceutical diuretics to facilitate the process [8, 19–21, 34, 35]. Bodybuilders may also employ these strategies to drop down to lower weight classes, which can provide a competitive advantage if the competitor is able to regain some of weight in the form of intramyocellular volume (“filling out” via glycogen and/or intramyocellular triglyceride storage) prior to competition. Although water and sodium are two separate dietary components, it is critical to comprehend that manipulation of one variable influences the other; hence, we will review these two variables together.

In a previously referenced survey on peak week and competition day strategies used by natural bodybuilders, water manipulation was the second most popular strategy implemented (behind carbohydrate manipulation) [11]. Researchers reported that competitors implemented water loading (65.4 %), water restriction (32.1 %), or both (25 %) to achieve a “dry” look. The amount of water consumed during the loading phase ranged from 4 to 12 L per day and was typically followed by water restriction 10–24 h prior to the competition. In addition to water manipulation, researchers also reported that competitors utilized sodium restriction (13.6 %), sodium loading (18.5 %), or both (6.2 %) with no consistent temporal order for the sodium loading/restriction regimen; however, sodium manipulation was generally practiced three to four days prior to competing. The use of dandelion tea was also reported due to its purported diuretic properties (see dietary supplementation section below).

In the previously discussed study by Mitchell et al. [14], researchers reported that 100 % of participants (n = 7) utilized the practice of water loading and water cutting during peak week. This strategy involved drinking > 10 L of water per day early in the week and then reducing the intake each subsequent day leading up to the competition. The theory behind this practice was to consume superfluous amounts of water to naturally increase fluid excretion in an attempt to preferentially excrete subcutaneous water; however, the participants reported that the results of this strategy were largely ineffective [14]. Out of the seven participants that manipulated water during peak week, three (42.8 %) also manipulated sodium to help remove subcutaneous water [14]. They reported greatly increasing sodium intake for the first three days of peak week followed by a complete restriction of salt intake for the three days prior to the competition; however, results were inconsistent and the participants stated they would not manipulate sodium in the future [14]. Note that the participants’ unanimous decision to abandon these water and sodium manipulation strategies suggests they had likely neither performed nor refined them previously (e.g., as a trial run or during peak week for another competition).

Other research supports the findings of the aforementioned studies. Probert et al. conducted a survey of 382 competitive bodybuilders along with personal interviews of 30 of the participants and reported that bodybuilders frequently engaged in the practices of sodium depletion and dehydration in the days prior to competing [12]. Although participants acknowledged the risks of these strategies, they downplayed them as temporary but necessary practices [12]. Indeed, case reports document potentially life-threatening conditions due to extreme practices of water and sodium manipulation [19, 20]. In one case, a 35-year-old male bodybuilder reported to the emergency room after feeling weak, dizzy, and experiencing painful muscle cramps while posing during a bodybuilding competition; tests revealed peaked T-waves on the electrocardiogram (ECG), hyperkalemia (high potassium levels), hyponatremia (low blood sodium levels), water intoxication, and rhabdomyolysis [20]. The bodybuilder reported drinking 12 L of water per day for seven days prior to the competition along with 100 mg daily of spironolactone (a potassium-sparing prescription diuretic) and salt depletion for two days prior to competition; he was successfully treated, stabilized, and discharged [20]. In another case, a professional 26-year-old male bodybuilder was transported to the emergency room the day after a competition as a result of heart palpitations and an inability to stand due to difficulty in moving his extremities [19]. He reported oral intake of 2 × 80 mg of furosemide (a prescription loop-diuretic) 24- and 48 h before the competition with the goal to enhance muscle definition; he lost 5–6 kg of bodyweight due to nocturia [19]. Tests revealed severe hypokalemia (low potassium levels; as opposed to hyperkalemia in the previously discussed case study likely due to the use of a loop-diuretic vs. a potassium sparing diuretic), hyperglycemia (high blood glucose levels), hyperlactatemia (high blood lactate levels), and sinus tachycardia with pronounced U waves on ECG consistent with hypokalemia [19]. Although hypokalemia is a potentially life-threatening condition, the bodybuilder was treated successfully and discharged the next morning [19].

Despite the various strategies reported by bodybuilders to manipulate water and sodium for the purposes of looking “full and dry,” current evidence does not indicate that these practices are specifically effective and/or safe. Additionally, although several water and sodium manipulation strategies have been published by a number of bodybuilding coaches who have worked with highly successful bodybuilders [16, 17, 36], neither the efficacy nor safety of these varying methodologies have been scientifically evaluated. Hence, physiological principles of body fluid regulation must be considered when attempting to formulate strategies to promote a “full and dry” appearance, and these strategies may be discordant with those currently used by bodybuilders and/or suggested by their coaches.

Total body water (TBW) content accounts for approximately 60 % of an average person’s body weight and is made up of intracellular water (ICW) (~ 67 %) and extracellular water (ECW) (~ 33 %). ECW is further compartmentalized into interstitial fluid that surrounds the cells (~ 25 %) and blood plasma (~ 8 %) [37, 38]. Hence, from a bodybuilder’s perspective, minimizing the extracellular interstitial fluid that surrounds the myocytes, specifically subcutaneous water, while preserving or increasing the intramyocellular ICW represents the ideal scenario for a “full and dry” appearance, i.e., whereby the appearance of muscularity is maximized. While this concept may seem like a simple task to accomplish by manipulating water and sodium alone, other strategies focused on optimizing intramyocellular volume (i.e., those targeting intramyocellular glycogen, triglyceride, and potassium content) may need to be considered along with the manipulation of water and sodium for the appearance of muscularity to be enhanced.

During normal fluid-electrolyte homeostasis, the extracellular compartment contains most of the sodium (Na+), chloride (Cl^−^), and bicarbonate (HCO_3_^−^), whereas the intracellular compartment contains most of the water, potassium (K+), and phosphate (PO_4_^3−^) [39]. Although both compartments contain all of the aforementioned compounds, the quantity of each varies between the compartments such that the total concentration of solutes (osmolarity) is the same [39]. Homeostatic mechanisms control water and electrolyte balance to ensure TBW and total body osmolarity (TBO) remain balanced and water redistributes itself between the intracellular and extracellular compartments such that the osmolarity of bodily fluids approximates TBO [37]. Indeed, Costill et al. investigated muscle water and electrolyte losses while participants cycled in a hot environmental chamber to lose 2.2 % (stage 1), 4.1 % (stage 2), and 5.8 % (stage 3) of their body weight over an estimated 5.5 h period [40]. When participants lost 2.2 % of their body weight within the first ~ 1.5 h in stage 1, 30 % of the water lost was ICW while 70 % was ECW [40]. However, the ratio of ICW to ECW lost became 52 % ICW/48 % ECW at stage 2 (~ 3.5 h mark) and 50 % ICW/50 % ECW at stage 3 (~ 5.5 h mark) [40]. The authors stated that the large loss of ICW in muscle at stage 1 may be explained by the significant loss of muscle glycogen content (which contains water) from pre-dehydration at 115 mmol/kg down to 76 mmol/kg; however, muscle glycogen content levels dropped at a much lower rate to 73 mmol/kg at stage 2 and 61 mmol/kg at stage 3 as the ratio or ICW:ECW stabilized [40]. Hence, the ratio of ECW to ICW loss appears to stay close to 1:1 as glycogen levels stabilize over time and higher levels of dehydration are reached. Thus, it seems that retention of muscle glycogen, by avoiding exercise that relies heavily on glycogen usage, may be important if methods of water loss are to effect a favorable loss of ECW relative to ICW (ECW > ICW) such that muscle size is retained while interstitial ECW is preferentially lost, enhancing the appearance of muscle “definition.” Relatedly, storage and retention of muscle glycogen is highly dependent on potassium availability (a primary intracellular cation - see above) [41–46], so ensuring adequate potassium intake during both carbohydrate loading and dehydration procedures seems paramount to optimizing stage appearance.

Importantly, if alterations in plasma osmolarity (via changes in total body water and electrolytes) reach a physiological threshold, then a complex neuroendocrinological network throughout the body in the brain, blood vessels, kidneys, and endocrine glands will respond to stabilize it [47]. Plasma osmolarity is affected by changes (increase or decrease) in the concentration of solutes (i.e., sodium) in the blood as well as changes in fluid volume; fluid volume is affected by total body water (TBW) [48]. Plasma osmolarity can increase by an excessive loss of water or a significant increase in sodium intake; conversely, plasma osmolarity can decrease with insufficient electrolyte consumption or excessive water intake [49]. Plasma osmolarity and blood pressure are regulated such that increasing plasma osmolarity results in decreased blood pressure and vice versa [49]. Additionally, blood pressure changes mediated by shifting plasma osmolarity are countered by arterial and renal baroreceptors [50].

During dehydration, as might be employed during peak week, plasma osmolarity increases, blood pressure decreases, and the renal baroreceptors in the juxtaglomerular apparatus (JGA) release the hormone renin; in turn, this activates the renin-angiotensin-aldosterone system (RAAS) [51]. When RAAS is activated, the process of maintaining fluid, electrolyte, and blood pressure homeostasis is initiated [51] and eventually releases the hormone aldosterone from the adrenal glands to further fine tune homeostasis [52, 53]. The baroreceptors in the aorta and carotid arteries also detect a decrease in blood pressure and signal the release of antidiuretic hormone (ADH, also known as vasopressin) from the pituitary gland to conserve water, increase blood volume, and increase blood pressure [48]. Conversely, if blood pressure increases due to increased arterial blood volume, the heart atria sense a stretch and release the hormone atrial natriuretic factor (ANF) to increase sodium excretion, inhibit renal vasoconstriction, attenuate renin secretion, and ultimately decrease blood volume and blood pressure [54].

 Collectively, if water and sodium are not carefully manipulated and timed, these physiological mechanisms that work to keep the body in homeostasis may not bring about the desired effect of selectively reducing fluid in the extracellular/subcutaneous space. Although these mechanisms are in place to keep the body in balance, not all hormones released have an immediate effect on the body when plasma osmolarity is altered. For example, a study displayed a delayed effect of ADH when investigators examined the effects of water loading on acute weight loss in combat sport athletes by comparing a water loading strategy for three days where the experimental group consumed 100 ml/kg/day of water compared to a control group that consumed 40ml/kg/day of water [55]. During the subsequent day of dehydration with both groups consuming 15ml/kg/day of water, the ADH levels in the water loading group rose from ~ 2.3 pmol/L to ~ 3.8 pmol/L at the 13th hour and ~ 5 pmol/L at the 24th hour of fluid restriction, at which time body mass losses exceeded those of the control group by 0.6 % (~ 2.5 vs. 3.1 % compared to baseline) [55]. Thus, despite the increased total fluid output from 3 days of water loading combined with one day of drastic fluid restriction, ADH levels were still climbing over 24 h of dehydration [55]. In another study, investigators reduced sodium intake to extremely low levels (10 meq/day) for ~ 6 days in 16 healthy men and measured the RAAS, plasma aldosterone, urine sodium, and serum sodium levels at 24 h, 48 h, and ~ 6 days after the intervention [53]. Although serum sodium levels remained fairly consistent between 137.6 and 139 meq/l for the ~ 6 day period, researchers reported that the RAAS activation was evident within 24 h and decreased urine sodium output from 217 meq/24 hr down to 105 meq/24 hr [53]. Furthermore, it took 48 h to observe a sharp increase in plasma aldosterone levels to further decrease urine sodium output to 59 meq/24 hr and another ~ 4 days for urine sodium output to stabilize at 9.9 meq/24 hr [53]. Hence, there is a temporal lag in establishing fluid and electrolyte homeostasis during which water and sodium manipulation may be implemented to induce diuresis before the protective homeostatic mechanisms fully manifest to halt water loss.

While bodybuilders often manipulate water and/or sodium by altering their intake [8, 11, 12, 14, 19, 20], another viable strategy may also be considered to increase diuresis. The literature on disuse atrophy and cardiovascular adaptations to weightlessness during space flight [56] reveals a previously described strategy [36] that physique competitors may employ to promote diuresis during the ~ 24 h before competition. Resting and/or sleeping with a “head down tilt” (HDT) position (typically − 4 to -6˚ whereby the entire sleeping surface is downsloping [57, 58] simulates the increase in cardiac venous return (and loss of orthostatic pressure) that occurs during microgravity. This results in diuresis and cardiovascular responses similar to those observed acutely during space flight [57, 59], mediated in part by an increase in atrial natriuretic peptide (released from the heart) and reduced plasma renin [60, 61]. Mauran et al., for instance, demonstrated that these hormonal responses and the associated diuresis and natriuresis return to baseline within 24 h [62], evoking a body weight loss of approximately 1.0-1.3 kg without changes in resting heart rate or blood pressure [58, 60, 61]. Short periods of more severe HDT up to -30 % evoke graded increases in central venous pressure beyond those of -6 % HDT [63], although the diuretic responses to HDT angle less than − 6 % have not been studied to our knowledge. Brief (≤ 2 h) periods of HDT up to -40˚ seem well tolerated [64, 65], but prolonged HDT at angles ≤ -12 % increases intracranial and intraocular pressure significantly [66]. Additionally, sufferers of gastric reflux should be aware that HDT could conceivably worsen symptomatology, given that elevating the head above bed level (the opposite of HDT) is an effective remedy [67–70]. This is likely not an issue for those who do not normally experience gastric reflux [71]. Thus, competitors could conceivably employ HDT when resting and sleeping during the 12-24 h before competition to further encourage diuresis as needed.

Another consideration when manipulating water and sodium intake is the important roles they play in carbohydrate absorption. Sodium-glucose dependent cotransporters (SGLTs) are proteins found in the small intestine that allow for glucose to be transported across the cell membrane; strong evidence suggests that the delivery of carbohydrate transport is limited by the transport capacity of SGLT1 [72–75]. Since carbohydrate loading appears to have potential benefits for bodybuilders to appear “full,” availability of sodium for co-transport of glucose across the cell membranes is important. Interestingly, the study by de Moraes et al. reported that carb loading induced various gastrointestinal symptoms in competitive bodybuilders [15]. Although sodium intake was not reported in this study, some of the symptoms may have been due to a lack of dietary sodium since bodybuilders have reported minimizing sodium intake as they approach contest day [11, 14, 20]. Additionally, since each gram of glycogen draws ~ 3–4 g water into the muscle [31] and this is a potassium dependent process (see above), a lack of water and potassium intake may also reduce the effectiveness of attaining a “full” appearance.

Contrary to the typical goal of reducing (extracellular, subcutaneous) body water, psychological disturbance/emotional stress can cause the retention of body fluids [76] via the actions of catecholamines (particularly dopamine) [77–79] and adrenocortical hormones, including both cortisol [80] and aldosterone [81]. Water retention during experimental stressful conditions requiring competition is subject to inter-individual variability, perhaps due in part genetic differences [82]. In extreme cases, emotionally stressful situations can evoke polydipsia and alter fluid homeostasis such that increases of up to 9 kg (~ 20lb) of body mass can accrue in as little as 48 h [78, 79]. Thus, there is support for the common empirical observation that psychological stress may counteract the competitive bodybuilder’s attempts to reduce body water, especially in extreme cases of pre-competition anxiety. The authors recommend performing a practice run of the peak week strategy ~ 2–4 weeks before the actual competition, in part to reduce anxiety and assure the competitor that the peak week strategy is both manageable and effective. Although beyond the scope of this article, stress management is acknowledged as an important aspect of sport psychology [83, 84] and is very likely important for competitors who find the final days before competition so stressful that it negatively affects on-stage appearance.

Based on these principles of water-electrolyte balance and the current evidence available, it appears that the manipulation of water and sodium should be carefully considered, planned, and practiced in conjunction with carbohydrate manipulation if they are to be utilized. While there appear to be some potential benefits to implementing these strategies to enhance the competition-day physique, potentially detrimental effects may occur if these variables are miscalculated and/or mistimed that may cause bodybuilders to miss their peak and/or incur health problems; thus, leaving these variables alone may be a better option for some competitors. Since bodybuilders have been reported to view sodium and water manipulation as temporary but necessary practices while downplaying the potential risks involved, caution must be practiced as extreme measures have been reported that led to life-threatening conditions [12, 19, 20]. The practical applications sections of this article will further outline how these variables may be safely manipulated based on the current evidence available.

### Dietary Fat

In addition to glycogen, muscle cells also store energy as intramuscular triglycerides (IMT). Indeed, nearly as much energy is stored in muscle cells as IMT as is stored as glycogen [85]. However, IMT stores vary considerably in humans, in part as a function of training status, muscle fiber type, insulin sensitivity, gender, and diet [85]. IMT may amount to ~ 1 % of muscle weight [86, 87], but because fat is less dense than skeletal muscle [88], the volume of IMT in a fully “fat-loaded” muscle cell could exceed 2 % of muscle volume [89, 90]. In rats (17), a single exercise bout can decrease muscle IMT content by 30 %, and three days of a high fat diet can boost IMT storage by ~ 60 % above baseline [91]. In humans, the dietary replenishment of IMT may be slower when glycogen restoration is also a priority [89, 92–94]. Still, IMT stores are increased by dietary fat intake [91, 95] and reduced during resistance [96] and endurance exercise [85].

Although fat loading has been a known strategy in bodybuilding circles for many years [97, 98], to our knowledge the strategy has not been studied directly in a bodybuilding peak week context (e.g., in combination with other dietary strategies such as glycogen supercompensation). In the rodent study mentioned above [91], three days of a high fat diet followed by three days of a high carbohydrate (CHO) diet resulted in supercompensation of both IMT and glycogen; however, 6 days of only a high CHO produced the expected glycogen loading effect but failed to elevate IMT levels above baseline. In humans, high CHO/low fat diets may actually precipitate a reduction of IMT stores [92–94], perhaps because IMT is used preferentially to cover the energetic costs of post-exercise cellular repair and glycogen-glycogenin assembly [94, 99]. Considering that a large (e.g., heavyweight male) bodybuilder may carry over 60 kg of muscle [100, 101], increasing IMT stores from a relatively “depleted” to a “loaded” state could conceivably increase muscle volume by > 1 % [85]; hypothetically, this translates to adding ≥ 0.6 kg of fat free mass. Hence, fat loading appears to be a promising strategy to be used in conjunction with CHO loading during peak week for bodybuilders, and thus warrants future study in a controlled setting.

### Dietary Protein

In conjunction with carbohydrate and fat intake during peak week, optimizing protein intake warrants discussion, as it is a major and indispensable component of the diet. The U.S. Recommended Dietary Allowance (RDA) for protein for adults is 0.8 g/kg [102], and has remained unchanged since ~ 1980, despite ongoing exposure of its inadequacy. In a call to re-evaluate and revise the RDA, Layman [103] contended that protein requirements are inversely proportional to energy intake. The latter point applies to dieters in general, but it has special significance for athletes in prolonged hypocaloric conditions, epitomized by pre-contest physique competitors. In light of mounting evidence, a daily intake of 1.2–1.6 g/kg has been proposed as optimal for the general population aiming to optimize health and longevity within a physically active lifestyle [104]. Toward the more athletic end of the spectrum, in the most comprehensive meta-analysis of its kind, Morton et al. [105] found that a protein intake of ~ 1.6 g/kg (upper 95 % CI of 2.2 g/kg) maximized muscle hypertrophy and strength in non-dieting recreational resistance trainees. In a study more representative of bodybuilders, Bandegan et al. [106] assessed whole-body protein synthesis via the indicator amino acid oxidation (IAAO) method and determined an Estimated Average Requirement of 1.7 g/kg/d with an upper 95 % confidence interval of 2.2 g/kg/d to be near their maximal attainable muscularity. In a similar protocol using the IAAO method, Mazzulla et al. [107] estimated the protein requirements of resistance-trained men to be 2.0-2.38 g/kg.

A systematic review by Helms et al. [108] reported that 2.3–3.1 g/kg of fat-free mass (FFM) was appropriate for resistance-trained subjects in hypocaloric conditions. However, out of the six studies included in the review, only two involved highly-trained competitive athletes, and only one study examined competitive bodybuilders. The latter study was conducted by Mäestu et al. [109], who tracked the body composition and hormonal profile of national and international level bodybuilders during the final 11 weeks of contest preparation. The competitors self-reported being steroid-free for a minimum of two years prior to the study. Protein intake was 2.68 g/kg (2.97 g/kg FFM) at baseline and 2.48 g/kg (2.66 g/kg FFM) at the final assessment point (3 days pre-contest).

Chappell et al. [2] reported that in high-level drug-free bodybuilders, end-of-preparation protein intakes of men and women who placed in the top-5 were 3.3 g/kg and 2.8 g/kg, respectively. Body composition was not reported in this study. Based on typical body fat percent ranges at the end of preparation, adding 4–6 % to the men’s intake and 13–15 % to the women’s intake would provide an estimate of grams of protein consumed per kg of FFM. A case study by Kistler et al. [3] on a high-level drug-free champion bodybuilder reported a protein intake of 3.4 g/kg (3.6 g/kg FFM). While the descriptive nature of these studies precludes the ability to draw inferences as to whether the observed level of intake was beneficial, neutral or detrimental from a physique standpoint, they appear to converge upon similar protein dosing in the final stage of the pre-contest period.

A potential consideration for protein dosing during peak week is whether to keep protein intake static or alter it during the carbohydrate depletion and loading phases. While no concrete evidence currently exists as to what is optimal to our knowledge, the study by de Moraes et al. [15] that reported an increase in muscle volume and enhanced physical appearance as a result of a carbohydrate-loading protocol provides some evidence that bodybuilders alter their protein intake during peak week. In this study, the depletion/loading protocol was three days of a low-carbohydrate (1.1 g/kg) and high-protein (3.2 g/kg) diet followed by only one day of a high-carbohydrate (9.0 g/kg) and low-protein (0.6 g/kg) diet. It seems likely that similar increases in muscle volume would have occurred if protein was kept static. However, despite the decreased protein intake (46.6 g on the carbohydrate-loading day versus 252.4 g on the carbohydrate depletion days), gastrointestinal distress was still significantly greater than the non-carb-loaded control group. This points to the possibility that keeping protein intake high during the loading day would have further worsened gastrointestinal symptoms, potentially due to excessive food intake. An alternative would be to keep protein static, but lessen the carbohydrate load (which in this case was ~ 714 g), allowing more than 1 day for carbohydrate loading. This seems a more practical approach (see above), such that an even greater total carbohydrate intake could be consumed but with less risk of gastrointestinal issues.

 A potentially viable strategy of altering protein intake during peak week is to keep protein intake relatively high at ~ 2.5–3.5 g/kg/day during the initial ~ 3 days of glycogen depletion portion of a glycogen super compensation strategy, followed by a relatively lower protein intake of ~ 1.6 g/kg/day during a high carbohydrate diet for 1–3 days (see above), finishing at least 24 h before the scheduled competition. Thereafter, a strategy for inducing diuresis and (further) elevating IMT stores during the day preceding competition by following a high protein, low carbohydrate (high fat) diet for a short period (~ 12–24 h) could be employed. As previously discussed, when carbohydrate loading using a low fat approach, IMT levels may decline, but elevated glycogen levels persist for several days in lieu of glycogen-reducing, demanding contractions (e.g., resistance exercise or excessive posing). High levels of intramuscular glycogen and the associated intracellular water would thus prevent the loss of ICW that typically accompanies diuresis. Increasing protein intake consumed the day before the show, or simply consuming protein at the high levels typically employed by pre-contest bodybuilders (~ 3.0–3.5 g/ kg / day; see above) and shown recently to be generally safe over longer periods [110], will encourage greater oxidative deamination of amino acids and ureagenesis [111] that approximate the maximal rates observed in healthy individuals [112, 113]. Clearance of blood urea in turn requires an osmotic gradient during its renal excretion, thus causing diuresis [114, 115]. Additionally, reverting back to a lower carbohydrate diet (e.g., one similar to that used early in the week to fat load in preparation for carbohydrate loading) would also promote loss of body water [116, 117]. Thus, increasing or maintaining a high protein intake while lowering carbohydrate and concomitantly increasing fat intake during the day before competing would reverse unwanted gains in extracellular/subcutaneous water experienced during carbohydrate loading [118]. It would also complement other strategic measures designed to induce diuresis such as manipulation of water/sodium/potassium intake, dietary supplementation, and body positioning (e.g., HDT) that would also afford a second opportunity for fat loading during peak week. Uncertainty of the effectiveness of modifying the de Moraes et al. and other protocols can only be mitigated by trial and error, as will be further discussed in the practical applications section, and warrants further scientific investigation.

### Dietary Supplementation

The consumption of sport supplements is common among bodybuilders and is often manipulated throughout their training phases (i.e. off-season, pre-contest) [2, 3, 5]. Although it is well understood that physique athletes utilize supplements such as protein powder, processed carbohydrates, pre-workout stimulants and ergogenic aids, creatine, vitamins/minerals, omega-3’s, thermogenics, diuretics and much more [2, 7], there is a paucity of data on how these supplements affect the athlete’s peaking process to enhance their physique. Hence, we will discuss the potential benefits of utilizing refined food supplements (i.e. protein/carbohydrate powders, fatty acids), creatine, and herbals during peak week.

Energy yielding food supplements like protein and carbohydrate have been regularly reported by other researchers examining bodybuilders [2, 3, 5]. Chappell et al. [2] examined fifty-one (35 male & 16 female) natural bodybuilders and found that ~ 75 % of males and ~ 89 % females supplemented with protein powders. Carbohydrate supplementation was less popular, with just ~ 37 % of the male competitors and no female competitor reporting their use. Bodybuilders may utilize these nutritional supplements as a means to manipulate and consume specific macronutrient quantities. As previously mentioned in the carbohydrate and water/sodium sections, bodybuilders seek to maximize muscle glycogen and its associated osmotic effect as a means to increase total muscle volume. Thus, it is common to supplement with various carbohydrate powders (e.g. dextrose, highly-branched cyclic dextrin, etc.). Carbohydrate characteristics such as osmolality, gastric clearance rate, and glycemic index are some of the variables physique athletes should take into consideration as these can significantly vary between sources and may impact gastrointestinal symptoms (e.g. bloating, cramping, diarrhea, constipation, etc.) [119–121]. Furthermore, the glycemic index of different carbohydrate sources have been shown to impact glycogen synthesis rates [122, 123]. This may be of greater importance for bodybuilders who are aiming to refill glycogen stores in a short time window (e.g. after making weight), as high glycemic carbohydrates have demonstrated superior glycogen resynthesis rates [122]. However, over a longer timeframe (i.e. 8 + hours), glycogen stores can be replenished similarly, regardless of feeding frequency [124], when consuming an adequate total amount of carbohydrates [125]. Additionally, data have demonstrated that combining protein with carbohydrates can enhance glycogen resynthesis [126]. However, it seems prudent that athletes do not “experiment” during peak week with new CHO, protein sources, or other supplements not integral to peak week specific strategies to reduce the risk of experiencing negative gastrointestinal symptoms or any other deleterious consequences.

There is substantial evidence supporting the use of creatine supplementation for bodybuilders. Chappell et al. reported that ~ 48 % of males and ~ 51 % of females supplemented with creatine during their contest preparation [2]. Creatine has been shown to improve body composition (i.e. increase lean body mass, decrease fat mass) [127, 128] and increase intracellular hydration status [129, 130]. Ziegenfuss et al. [129] demonstrated that a three day creatine loading phase increased intracellular fluid volume by ~ 3 % without impacting extracellular fluid. The use of multifrequency bioelectrical impedance analysis (MBIA) caused some to initially interpret the data with some skepticism. However, a follow-up study employing the same three day creatine loading scheme observed a 6.6 % increase in thigh muscle volume among elite NCAA power athletes as determined by the gold-standard magnetic resonance imaging [131]. Creatine supplementation has also been shown to aid in glycogen synthesis and supercompensation [132]. Additionally, consuming CHO with creatine increases creatine loading [133], which increases cellular hydration as noted above [32, 129]. Finally, muscle creatine levels decline very slowly after loading [134], so creatine intake after peak week glycogen loading is not needed except perhaps in small amounts to potentially accelerate last minute, competition day carbohydrate delivery into skeletal muscle. Thus, creatine supplementation may be a potentially effective tool during peak-week for acutely expanding muscle size. However, it should be noted that not all individuals will respond to exogenous creatine intake vis-à-vis significantly increasing muscle creatine content [135, 136]. In particular, “responders” tend to be those who have a larger type II muscle fiber area (i.e., those with an innate proclivity for sprinting and/or strength/power sports) [137, 138] and/or those with lower initial creatine levels, perhaps due to lack of intake (e.g., those who have not been supplementing with creatine or who are non-supplementing vegetarians) [139].

Omega-3 fatty acid [eicosapentaenoic acid (EPA), docosahexaenoic acid (DHA)] supplementation has also been observed in bodybuilders [2, 3]. Chappell et al. reported that 39 % of males and 47 % of females consumed an omega-3 supplement (e.g. fish, krill, flax-oil) [2]. Although substantial data in many population demographics supports the use of EPA & DHA as a means to reduce systemic inflammation and improve insulin sensitivity [140, 141], it remains unknown if this can enhance the peaking process.

As discussed previously, the use of diuretics has been commonly reported in the competitive bodybuilding space [8, 19–21, 34, 35]. Bodybuilders often use diuretics (both herbal and synthetic drugs) to increase urine output and excrete sodium in an effort to alter fluid volume, enhance body composition, and present a more aesthetic physique [142]. Moreover, some may use diuretics to reduce total body mass with the aim to make a specific weight class [8, 19–21, 34, 35, 143]. For example, Caldwell et al. [143] investigated the effects of a prescription diuretic (furosemide 1.7 mg/kg) on athletes of various sports (e.g., weightlifters and martial artists) and reported a significant reduction in total body mass (-3.1 ± 0.8 kg) over a 24-hour period. However, due to the potential health dangers and their ability to mask the use of performance enhancing drugs, prescription diuretics have been banned by the World Anti-Doping Agency [144]. While these drugs are presumably not used by natural bodybuilders, they have been employed by enhanced non-tested competitors [19, 20]. Interestingly, some herbal supplements that are not banned have demonstrated a diuretic effect and may be employed by enhanced and natural bodybuilders alike. For example, *taraxacum officinale* (dandelion) has been shown to significantly increase urine frequency and excretion output in an acute fashion (i.e. within a 10 h window) [145]; however, to our knowledge, no research has directly examined its impact on intracellular vs. extracellular fluid shifts or on its effectiveness during peak week.

Vitamin C (ascorbic acid) is water-soluble and considered non-toxic even in high amounts [146]. Since it requires renal filtration for excretion, it also brings about osmotic diuresis [147]. Research supports a diuretic effect of both oral and IV vitamin C [148], with daily doses as low as 11 mg/kg producing diuresis in children [149], although a 500 mg IV dose failed to induce diuresis in adult males [150]. A study of both healthy subjects and vitamin C deficient patients demonstrated that urinary Vitamin C losses (and accompanying diuresis) occurs only above threshold blood concentrations of ~ 14 mg/L (which corresponds to tissue saturation levels). These data suggest that reaching diuresis-promoting vitamin C blood concentrations varies as a function of rates of absorption and uptake/deposition into tissues [151] (3). Given its common usage, relative safety, and potential effectiveness as a non-pharmacologic diuretic, the use of ascorbic acid in a peak week scenario (including dosing patterns to minimize GI distress and optimize blood concentrations in the context of meal timing and other factors that may influence absorption) warrants of further research. Indeed, due to the paucity of research available on the subject, it is difficult to make definitive recommendations on usage and dosage during peak week. However, based on the evidence available, repeated dosing (every few hours) of 500–1000 mg of vitamin C is a viable strategy to be utilized during the 12–24 h before competitive stage appearance to potentially accelerate body water loss with minimal side effects (e.g., gastrointestinal distress). Please note that caution is warranted as excessive vitamin C consumption may cause osmotic diarrhea [152].

Caffeine use is another supplement of special mention due to its diuretic properties. Doses of at least ~ 250-300 mg caffeine (2–3 cups of coffee) can be taken to promote diuresis acutely in those who are not caffeine-tolerant due to chronic use [153]. On the other hand, several days of abstinence can restore sensitivity to caffeine’s diuretic effects (although the diuretic effect is still only present at these larger doses) [154]. Caffeine’s diuretic, mood-improving [155] and performance enhancing effects [156] should also be considered in the context of potential sleep disturbances if taken acutely to promote diuresis to make weight and/or the night before competition, as well as withdrawal effect if use is abruptly discontinued [157]. One potential peak week strategy would be to limit caffeine early in the peak week process (especially in chronic users, to restore sensitivity), employ it early in the day as a diuretic (e.g., on the day before competition) to limit adverse effects on sleep quality, and continue its use thereafter (e.g., upon rising the day of competition) to prevent withdrawal effects on both fluid homeostasis and/or mood and arousal [157]. It has been noted that caffeine can be employed (3-8 mg/kg) as an agent to speed glycogen loading [158], although data are sparse and equivocal as to this effect [159]. Thus, athletes who might choose to include caffeine to enhance carbohydrate loading in the middle of the peak week may potentially also be forfeiting its usefulness as a diuretic during the days thereafter (i.e., when “drying out” the ~ 24 h before stepping on stage).

### Fiber and FODMAPs

Dietary fiber is indigestible plant matter of carbohydrate sources that can be categorized as water-soluble or insoluble (i.e. fermentable) and plays a vital role in gastrointestinal health and bowel-movement regularity [160]. Bodybuilders who are aiming to reduce total body mass during peak week as a means to make a particular weight class may benefit by intentionally reducing fiber intake. For example, Reale et al. [55] investigated the effect of dietary manipulations (i.e. macronutrient, fiber, sodium, and water intake) on acute weight loss for combat athletes and prescribed 10-13 g of fiber to reduce total gut content and body mass. Different food sources impact fecal bulking characteristics and those high in fiber tend to increase water in the interstitial space and stool bulk [161]. Data have shown a direct relationship between fiber intake and bowel contents with acute restriction periods (as short as two days) to be effective at emptying/clearing the gastrointestinal tract [162]. Thus, the rationale to reduce fiber intake before the competition is typically to minimize the risk of bloating/water retention [11], and for some, may be an effective strategy for making a weight class.

Although research is limited on the topic, Chappell et al. [11] reported that the bodybuilders they observed severely reduced their fiber intake primarily by reducing/omitting fibrous vegetables during peak week. Additionally, it is well understood that fermented oligosaccharides, disaccharides, monosaccharides, and polyols (FODMAPs) are poorly absorbed, draw fluid within the GI tract, and increase the likelihood of bloating/gas [163]. Thus, it may be advisable for bodybuilders to limit high FODMAP food sources during peak week. This may be one reason why dairy/lactose and gluten rich food sources are also anecdotally restricted in this period as well. On the other hand, fiber sources such as guar gum [164] and psyllium [165], which have been shown to reduce symptoms of irritable bowel syndrome dominated by both constipation and diarrhea, might be employed on an individual basis to offset gastrointestinal distress, as noted above in the study by de Moraes et al. [15]. Despite the lack of data within this demographic, dietary fiber is likely a variable that can impact a bodybuilder’s peaking process and should be considered on an individual basis in context with the other aspects of the peak week approach.

### Training

Since bodybuilders invariably train primarily with resistance exercise (RE), the extent to which RE in particular reduces glycogen and IMT warrants consideration. In an early study of fuel use during RE in trained bodybuilders, Essen-Gustavsson and Tesch [96] found that a high volume, lower body RE session reduced both vastus lateralis glycogen and IMT by ~ 30 %, and that both resting levels and the extent of depletion correlated to the energetically-related enzymes hexokinase and 3-hydroxy-Co-A dehydrogenase, respectively. In another study, just three sets of arm curls (80 % 1RM or ~ 12RM) was enough to reduce biceps brachii glycogen by 24 % and elevate muscle lactate ~ 20 fold in trained bodybuilders [166]. Similarly, Robergs et al. [167] found that 6 sets of knee extensions (~ 13 reps/set; 2 min rest intervals) reduced muscle glycogen by approximately 40 % in resistance trained males, but glycogen levels recovered 50 % of losses during 2 h of fasting rest, presumably due to the immediate post-exercise assimilation of glycogenolytic metabolites (e.g., lactate) [168]. The same group also found that an external workload-matched regime (employing double the load such that sets averaged only ~ 6 reps to failure) produced a nearly identical pattern of glycogen use and immediate post-exercise recovery. Thus, RE performed with commonly employed rep ranges among bodybuilders substantially reduces muscle glycogen stores in a manner related to the workload/volume of a given bout.

In line with previous research suggesting that fat oxidation is greater in females as well as those with higher body fat levels [85, 169], a study of untrained obese women found that 42 % of resting mixed muscle IMT stores were used during only 6 sets of 10 repetitions of knee extension [170]. While IMT had returned to 33 % below baseline 2 h after exercise despite no intake of food, muscle glycogen stores diminished by only 25 % over the course of the bout but failed to significantly recover in the absence of food consumption [170]. The above data suggest that IMT restoration may proceed slowly in lieu of dietary sources [171], whereas CHO is required to substantially restore glycogen levels beyond an acute post resistance training re-sequestration of glycolytic intermediates.

Thus, the potential to modify intramuscular glycogen and IMT stores via diet (see above) and exercise is clear, but the corresponding effects may be variable across bodybuilders as a function of pre-contest diet (macronutrient composition and content may affect resting stores), muscle enzyme activity, and gender, among other uninvestigated variables. Exercise-induced muscle damage may also be important in interpreting the above data since it is highly variable [172–174], a function of training status [175], and known to impair muscle insulin sensitivity [176] as well as glycogen replenishment [177]. Avoiding excessive muscle damage may thus be important when considering a resistance training strategy during peak week not only to maximize glycogen and IMT stores, but also to prevent unwanted delayed onset muscle soreness that may impede the ability to activate muscles [178] during posing. In fact, the energetic demands of recovery from a damaging bout may be so great in extreme cases that glycogen levels can continue to decrease post-exercise and not fully recover in 24 h despite high CHO consumption (10 g/kg/day) [179]. Variability in the extent of post-exercise inflammation [180, 181] may also explain the above-noted variability in the extent of hydration that accompanies glycogen loading. Resting IMT and glycogen levels are higher and used more readily in trained subjects who employ greater absolute workload. However, post-exercise restoration of both fuel sources correlates with insulin sensitivity and proceeds similarly relative to resting stores regardless of training status [182]. Thus, the high insulin sensitivity generally observed in pre-contest bodybuilders [5, 7, 90, 183, 184] confers an advantage for IMT and glycogen restoration after high substrate-demanding training sessions [185], but their greater muscle mass and capacity to reduce muscle fuel stores dictate that dietary fat and CHO intake must be commensurately large to ensure a super compensatory effect.

### Practical Applications for Peak Week

It is evident that bodybuilders implement a variety of peak week strategies despite the paucity of bodybuilder-specific research on safety and efficacy. Since there are many interrelated variables to consider during the peaking process that directly influence each other, specific peak week recommendations are not possible. Furthermore, there are significant inter-individual responses to the manipulation of these variables and bodybuilders may have to take different approaches during peak week depending on their circumstances, goals, and how their body responds to the alterations of the variables. For example, peak week approaches could differ substantially based on their circumstances of a bodybuilder that needs to make a weight for a specific weight class as compared to a bodybuilder that is not bound by a weight limit. Similarly, different approaches might need to be implemented by athletes competing in the various subdivisions of bodybuilding (i.e., women’s physique/figure/wellness/bikini/fitness and men’s physique/classic physique) where judging standards may differ from those of traditional bodybuilding.

While an in-depth discussion of the nuanced and somewhat fluid judging standards (which vary across the numerous bodybuilding federations/organization) of the various competitive physique divisions is beyond the scope of this review, the following general considerations can be applied in constructing a peak week strategy for these other divisions: (1) The standard for leanness in the non-bodybuilding women’s divisions often call for higher body fat levels and less muscularity than women’s bodybuilding, and may also thus require few or none of the peak week manipulations described here; (2) Anecdotally, female competitors (typically in Bikini or Figure divisions) may intentionally reduce total body fat to obtain competitive lower body fat levels and, rather than applying diuretic procedures, will “water load” in an attempt to reduce the appearance of excessive leanness but retain the desired appearance of more evenly distributed body fat distribution, and; (3) Fitness competitors, where physical performance as well as physique appearance are judged, may have to create highly individualized approaches to water and fuel restoration that optimize competitiveness, minimize injury risk, and account for the relative timing of routine and physique rounds over the course of a competition.

**Fig. 1:**
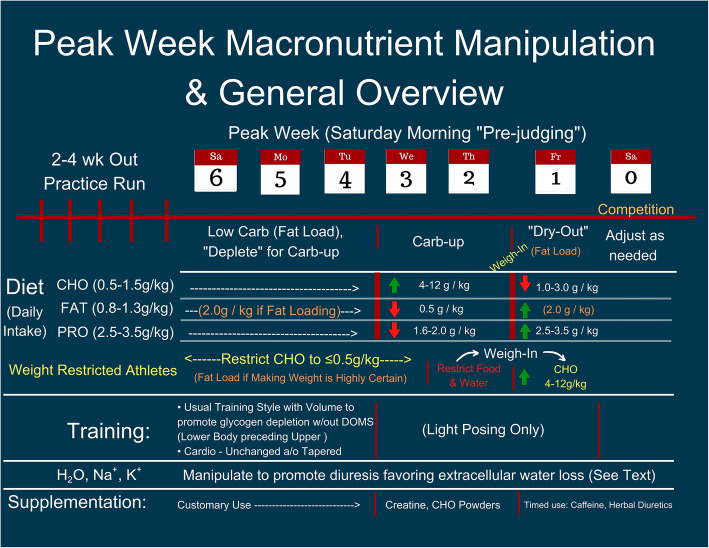
Peak Week Macronutrient Manipulation and General Overview

Given the current evidence discussed within the body of the manuscript, we offer the following general recommendations for bodybuilders to help readers develop individualized peak week strategies that coordinate macronutrient intake, hydration and electrolyte strategies, supplementation, and resistance/endurance exercise regimens. It is important to emphasize that these recommendations should not be considered concrete “rules” as there is significant individual variability of how athletes may respond to the manipulation of these variables. Indeed, due to the number of variables that may be manipulated and the virtually endless scenarios that may occur, we present more specific peaking guidelines for: (1) A women’s physique competitor (60 kg that is not bound by a weight limit (BB1); (2) a superheavyweight (105 kg) bodybuilder that is not bound by a weight limit (BB2); (3) a classic physique competitor that needs to be under a weight limit (85 kg) based on his height class (BB3). In all circumstances, it will be assumed that competitors check-in (and weigh-in if applicable) on Friday afternoon to compete on Saturday morning for prejudging and Saturday evening for finals. Please note that despite these specific circumstances, the recommendations presented in Fig. 1 and Tables 1, 2, and 3 should be seen as recommended starting points that will likely require adjustments based on the individual’s responses to the alteration of the variables. The mock peak week strategy in the Fig. 1 are presented only as illustrative examples and should not be considered prescriptive dietary, exercise and/or medical advice. ​Please refer to the text for a detailed rationale for the macronutrient, water, sodium and potassium manipulation presented in Fig. 1 and  Tables 1, 2 and 3. To these ends, peak week strategies would include the following considerations:

1. During a depletion/supercompensation resistance exercise protocol, resistance exercise should engage all major muscle groups and employ a variety of exercises to ensure widespread reduction in IMT and glycogen levels across the entire muscle mass.
2. Using a relatively high repetition scheme (> 12 repetitions) with either a lower or higher volume approach [167], and exerting enough effort and/or load to engage most fiber types [186–188] but stopping short of failure and employing a training volume/intensity taper while avoiding novel exercises seems prudent to ensure muscle damage is minimized.
3. Exercises that overload the muscle in their lengthened range/are eccentric dominant (e.g. Romanian Deadlift, DB Lat Pullover, DB Fly) should be minimized since training at lengthened positions has been shown to increase muscle damage [189].
4. Cardiovascular exercise should be tapered and preferentially eliminated before attempting to super compensate fuel stores dietarily in the days preceding competition.
5. Resistance training during peak week should generally take place early in the week, spread out over 3–4 days depending on the athlete’s accustomed training split, to allow adequate time for supercompensation during the days before stepping on the competition platform. Training legs first in this series of peak week workouts allows the greatest time for recovery in these muscle groups.
6. The potential for glycogen loading to impair IMT storage suggests that separating periods of glycogen and fat loading may be prudent, with a high CHO diet preceding efforts to fat load [92]. Reducing fat coingestion with large amounts of carbohydrate may also avoid negative effects of free-fatty acids on glycogen formation [190], reduce gastric distension by speeding gastric emptying, as well as improve glycogen loading by further elevating blood glucose and insulin levels [191–193]. When consumed on different days, diets containing fat at 2 g/kg/day [92] and CHO at 10 g/ kg/day [100] can restore and potentially super-compensate their respective fuel depots within 24 h. Individual variability and athlete goals/needs may require different strategies, including allowing > 24 h for glycogen loading [194] if circumstances permit.
7. Rather than introducing new foods, consuming mainly the same dietary constituents during peak week as those consumed during the preceding weeks/months beforehand may also be helpful in avoiding gastric distress. Since fruit and fructose sources of carbohydrates better stimulate liver glycogen restoration, whereas glucose does so for muscle glycogen [195], it is recommended that the majority of the carbohydrates consumed come from starchy/glucose-based sources. Of note, however, is that combinations of glucose, fructose, and sucrose with sports drinks have been shown to enhance the rate of fluid absorption from the proximal small intestine [196]. Thus, it is recommended that athletes experiment prior to peak week as to what carbohydrate sources work best for them.
8. Ensuring protein is co-ingested, albeit in perhaps lower quantities, with CHO may increase insulin release and facilitate glycogen loading [197, 198].
9. A higher protein intake (i.e., 3.0 g/kg) may be combined with a higher fat intake during periods of CHO depletion to initiate fat loading followed by CHO loading with a lower protein intake (i.e., 1.6 g/kg) to super compensate glycogen stores. Once carbohydrate loading is complete, a higher protein (3.0 g/kg)/high fat/lower CHO diet may be implemented. Once again, individual variability and athlete goals/needs may require different strategies to peak the physique.
10. Various CHO loading strategies have been reported in bodybuilding. For example, Roberts et al. [199] discuss the practice of front-loading CHO (intake is higher earlier in the week and then lowered to maintain muscle fullness leading to the competition) and back-loading CHO (intake occurs later in the week but may result in less time to make adjustments to the physique). Alternatively, a model whereby CHO is depleted early in the week (7 − 4 days out), loaded mid-week (3 − 2 days out), and then adjusted/maintained (1 day out) could also be utilized. In the study by de Moraes et al. [15], a back-loading method was used, but more evidence is required before more concrete recommendations are made. Based on the current evidence, we recommend the third model discussed, as presented in Table 1 for the 60 kg female physique competitor and the 105 kg male bodybuilder,  to gain the benefits of front-loading and back-loading; however, individual responses/preferences to CHO loading and the individual’s needs (i.e., making a weight class may require back-loading) must all be considered.

**Table 1:**
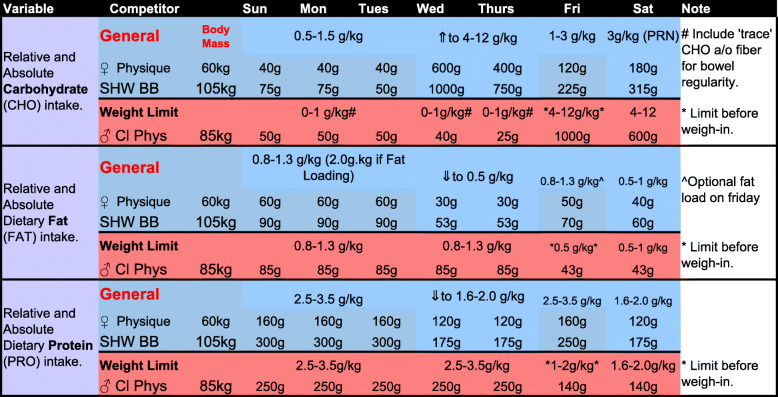
.
11. The preceding pre-contest diet may affect competitor tolerance to dietary manipulation, as well the extent of dietary restriction of fat and CHO during peak week training days needed to precipitate a subsequent super-compensatory effect. For instance, those competitors following a high CHO/low fat, but very low calorie diet (leaving glycogen levels chronically low) might best avoid completely eliminating CHO during peak week training. However, those who have been using a low carbohydrate approach could continue employing a low CHO diet during peak week but might be wary of excessive training (tapering instead approach) if glycogen levels are likely diminished at the start of peak week.
12. Generally speaking, lowering CHO and raising fat intake (as tolerable) during the peak week training (“depletion”) days may facilitate glycogen loading during the days after training, and simultaneously ensure IMT levels are not lowered excessively. After 1–2 days of glycogen loading mid/late week as recommended in our CHO loading approach, IMT levels could then be elevated the day before competition with a high fat/low CHO approach that would also serve to reduce body water [117]. Once again, individual variability and athlete goals/needs may require different strategies with these general guidelines.
13. The practice of water loading followed by water restriction has been documented to be a safe and effective weight loss strategy to lose TBW in combat athletes [55]. While the ratio of ECW to ICW lost was not reported in this study, Costill et al. [40] (as previously stated) reported that the ratio of ECW to ICW loss stays close to 1:1 when glycogen levels stabilize over time and higher levels of dehydration are reached. Thus, it seems that retention of muscle glycogen, by avoiding exercise that relies heavily on glycogen, may be important if methods of water loss are to effect a favorable loss of ECW relative to ICW (ECW > ICW) such that muscle size is retained while interstitial ECW is preferentially lost, potentially enhancing the appearance of muscle “definition.”
14. Many variables may alter the approach used to water load/water deplete (i.e., how much water the athlete is accustomed to drinking on a regular basis), but the participants in the Reale et al. study successfully lost TBW by drinking a large quantity of water (100 ml/kg) for three days followed by significantly cutting water to 15 ml/kg on the fourth day [55] with no deleterious effects. Alternatively, water intake can be kept relatively constant (with the exception of a few hours before competing to prevent any abdominal distention) to minimize the variables being manipulated; indeed, this might be the best approach if no practice runs are performed prior to competing.
15. Since muscle glycogen creates an osmotic effect, pulling water into the cell as glycogen is stored [26], CHO loading should be carried out in conjunction with water intake [199] so that muscle ICW can be maximized while CHO intake is high. After approximately three days of water loading with a higher CHO intake (if the water loading method is used), water intake can decrease to ~ 15 ml/kg for 24 h which will help to induce diuresis within the ~ 24 h prior to the competition. Note that this recommendation is based on what has been studied and reported; however, the authors recognize that higher water intakes may be preferential such as 30–40 ml/kg but have not been investigated and thus require further research.
16. Increasing or maintaining a high protein intake while lowering carbohydrate consumption and concomitantly increasing fat intake during the day before competing conceivably would reverse unwanted gains in extracellular/subcutaneous water experienced during carbohydrate loading [118].
17. Sodium intake has been reported to be significantly reduced by bodybuilders during peak week [11, 14, 20], but the timing of this practice should be carefully implemented and sodium intake should not be reduced simultaneously with CHO loading since evidence suggests that the delivery of CHO is limited by the transport capacity of SGLT1 [72–75]. Once CHO intake has decreased after glycogen loading, sodium intake may be temporarily reduced since research indicates that the RAAS activation is evident within 24 h and it takes ~ 48 h to observe a sharp increase in plasma aldosterone levels [53]. This temporal lag in establishing fluid and electrolyte homeostasis, if timed correctly, may be implemented to induce diuresis before the protective homeostatic mechanisms fully manifest to halt water loss. Depending on the bodybuilder’s needs prior to competition (e.g., necessity to make a weight class), various sodium intake scenarios are presented in Table 2. Alternatively, sodium can be kept as a constant to minimize the variables being manipulated; indeed, this might be the best approach if no practice runs are performed prior to competing.

**Table 2:**
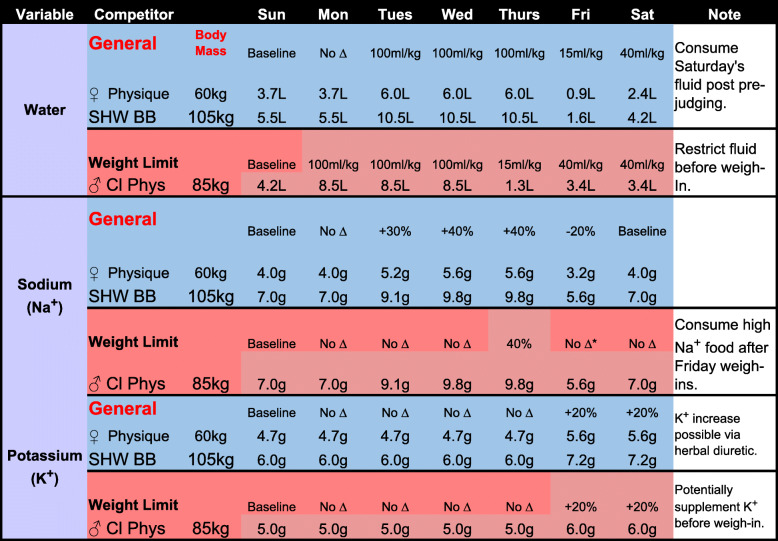
.
18. Storage and retention of muscle glycogen is highly dependent on potassium availability (a primary intracellular cation) [41–46]. Hence, ensuring adequate potassium intake during both carbohydrate loading and water cutting procedures (if implemented) is likely paramount to optimizing stage appearance via storage and retention of muscle glycogen and thus encouraging a more favorable loss of ECW relative to ICW when employing dehydration strategies.
19. Reducing fiber intake during peak week appears to offer some potential benefits. Rale et al. [55] reported that reducing fiber intake to 10-13 g/day for ~ 5 days successfully reduced total gut content and body mass in contact fighters. Data have shown a direct relationship between fiber intake and bowel contents with acute restriction periods (as short as two days) to be effective at emptying/clearing the gastrointestinal tract [162]. Thus, the rationale to reduce fiber intake before the competition is typically to minimize the risk of bloating/water retention [11], and for some, part of their process to make a weight class.
20. The utilization of some supplements during peak week may prove to be beneficial to athletes. Creatine supplementation has been shown to aid in glycogen synthesis and supercompensation [132]. Additionally, consuming CHO with creatine increases creatine loading [133], which increases intracellular hydration [32, 129]. In conjunction with creatine, carbohydrate powders (e.g. dextrose, highly-branched cyclic dextrin, etc.) may also be considered. Carbohydrate characteristics such as osmolality, gastric clearance rate, and glycemic index are some of the variables bodybuilders should take into consideration as these factors can significantly vary between sources and may impact gastrointestinal symptoms (e.g. bloating, cramping, diarrhea, constipation, etc.) [119–121]. Both hydrolyzed whey protein powders and carbohydrate powders may be utilized as means to manipulate and consume specific macronutrient quantities without having to consume larger volumes of food.
21. Emotionally stressful situations can evoke polydipsia and alter fluid homeostasis in as little as 48 h [78, 79]. Hence, psychological stress may counteract the competitive bodybuilder’s attempts to reduce body water, especially in extreme cases of pre-competition anxiety. As noted previously, we recommend performing a practice run of the peak week strategy ~ 2–4 weeks before the actual competition, in part to reduce anxiety and assure the competitor that the peak week strategy is both manageable and effective.
22. Resting and/or sleeping with a “head down tilt” (HDT) position (typically − 4 to -6˚ whereby the entire sleeping surface is downsloping [57, 58] simulates the increase in cardiac venous return (and loss of orthostatic pressure) that occurs during microgravity and results in diuresis and cardiovascular responses [57, 59]. Thus, competitors could conceivably employ HDT when resting and sleeping during the 12-24 h before competition to further encourage diuresis. This potential benefit should be balanced with possible detrimental effects of the practice on sleep patterns, which could interfere with competition performance.
23. Scale weight can be used during peak week to evaluate and confirm hydration levels (see Practical Considerations section below).
24. Since there are a multitude of variables involved and substantial biological inter-individuality, a practice or “mock” peak week performed during the ~ 2–4 weeks before competition can provide invaluable information regarding the appropriate extent and timing of alterations of diet and training during peak week. Furthermore, it may attenuate the levels of stress that a bodybuilder may have prior to competing, which may facilitate how the body responds to the peak week process.
25. Athletes who may be partaking in a series of competitions in relatively rapid succession, typically on a weekly basis, should construct peak week strategies (as in our examples here) that can be replicated, with additional adjustments as needed, during the time period between competitions. This may require competitors to maintain strict dietary control and rapidly establish fluid homeostasis post-competition so as to restore the baseline starting conditions (e.g., muscle glycogen levels) upon which a given peak week strategy may rely. Further, in addition to the medical risks noted previously, the ill-advised use of pharmacological diuretics during peak week may likely disrupt fluid homeostasis and diminish the reliability and thus success of diuretic strategies employed over a series of competitions in close temporal proximity.

**Table 3:** Supplement Considerations During Peak Week

Supplement	Notes on Strategic Supplement Use during Peak Week
**Creatine**	Creatine (Cr) supplementation has been shown to increase intracellular fluid volume by ~ 3 % without impacting ECF and up to 6.6 % for total muscle volume [131]. Additionally, it has been shown to aid in glycogen synthesis and super-compensation [31, 129]. Although it’s advisable for physique athletes to supplement with Cr for the entire contest preparation, continuing to supplement throughout peak week is suggested. If an athlete has supplemented with creatine throughout the contest preparation, a ‘maintenance’ dose of 0.03 g/kg/day may be employed. If the athlete has not regularly used Cr throughout the contest preparation, supplementation during peak week would require a ‘loading’ 0.3 g/kg/day. This would translate to 25 g/day during a loading phase and 5 g/day for maintenance when using a 180lb (82 kg) subject as an example.
**Carbohydrate Powders**	During the carbohydrate loading process with the intent of super-compensating muscle glycogen, carbohydrate supplements may be beneficial to reduce this risk of negative gastrointestinal symptoms (e.g. bloating). With carbohydrate intakes potentially reaching 10-12 g/kg, the sheer food volume alone can be problematic. Some carbohydrate supplements (e.g. highly branched cyclic dextrin) may be favorable due to their osmolality and gastric clearance rate. This may particularly advantageous for physique athletes who had to deplete and dehydrate to make weight for their class and have a small time window to carbohydrate load.
**Herbal Diuretics**	Herbal diuretics (e.g. dandelion root) may be employed in an acute fashion to increase urine frequency and excretion output [149]. The potassium rich herbs may further enhance the dehydration process, specifically by expelling extracellular water [150, 151]. As noted in the text, caffeine is also an effective diuretic in non-habituated users [152], but runs the risk of causing sleep disturbance.

It is essential to understand that none of the aforementioned peak week strategies will provide a physique makeover to compensate for a lack of preparation or adherence during the off-season or pre-contest phases of contest preparation. Body fat should be minimized ~ 2–3 weeks before competition, such that the competitor can focus on minimizing subcutaneous water to best display muscularity, and maximizing muscle size by increasing intramuscular stores of glycogen and triglyceride. Thus, employing peak week strategies is merely a means to achieve superior on-stage competition day appearance by “fine tuning” the body compared to simply maintaining the pre-contest diet and training strategies (i.e., those focused primarily on reducing body fat and maintaining or gaining muscle mass) .

### Practical Considerations for Day of Competition

Ideally the physique presented on stage represents the athlete’s best possible appearance, superseding that of the preceding weeks and months. Ensuring that the peak occurs on the day of competition often requires a tailored approach with at least the following considerations:

- Competition day schedule: When is the athlete judged and how many times? Many competitive organizations include multiple judging rounds [200–202] and categories such that the competition may transpire over the course of an entire day (or longer).
- Strategies (pre-planned or otherwise) to fine-tune the physique’s appearance on competition day by manipulating water, food, and dietary supplement intake as needed.
- Subjective appearance and perception of the physique (per the above) and other means of assessing stage readiness. Of course, the goals of peak week to minimize subcutaneous water and ensure IMTG and skeletal muscle glycogen stores are maximized putting the muscle bellies in full relief and displaying maximal “muscularity” should largely be accomplished before waking the day of competition. In bodybuilding vernacular, these components of muscularity could be considered “dryness” (lack of subcutaneous fluid) and “fullness” (the muscle cell fuel stores are fully repleted / super compensated). However, some fine tuning is often necessary to optimize the physique’s appearance when being judged.

To our knowledge, research examining the extent to which subjective or other practical means of ensuring bodybuilding competition day preparedness are associated with the presumed underlying fluid and histological measures has not been studied. However, the following are commonly accepted and previously suggested [36] ways of evaluating contest day readiness:

- Are the muscle glycogen stores “full,” and can the athlete get a pump? Glycolytic metabolites (e.g., lactate and inorganic phosphate) derived from glycogen use produce a post-exercise reactive hyperemia response known as a “pump” [203] that swells muscle tissue, increasing thickness as much as ~ 10 % [204, 205]. This poses an advantage for acutely increasing muscle size before stepping on stage and shifting fluid into specific muscle bellies (ideally even thereby reducing interstitial subcutaneous fluid volume to further enhance the appearance of muscularity), such than an athlete can preferentially “pump up” the musculature to improve the balance of the muscular development.
- Is the athlete “dry?” Has body water been reduced enough to minimize subcutaneous fluid to noticeably highlight the underlying musculature?
- Is the athlete “flat?” Creating a situation of muscle fullness and physique dryness requires a tight physiological balancing act. The hyperemic “pump” requires adequate body fluid to move into the muscle belly; however, an athlete with high muscle glycogen levels but excessively reduced body water may experience “flatness,” i.e., a lack of a muscle pump usually associated with a withered appearance due to excessive dehydration. On the other hand, lack of muscle glycogen to serve as the source for metabolic osmolytes for the pump effect [203] could also be to blame.

Both glycogen stores (“fullness”) and dehydration (“dryness”) are dependent upon rapidly changing fluid homeostasis. Thus, we propose that scale weight may be employed as a rudimentary, but practical and objective marker of body hydration (“dryness”) in the context of the muscle pump and visual appearance, as well as the urinary fluid losses [note that urine color is an adequate field measure of hydrate state, but may be altered by dietary supplement consumption [206, 207]. Thus, measuring body weight throughout peak week and its rate of change can help determine the extent to which body water has been minimized on the day of competition. Measurements for a hypothetical competitor are given in Table 4. We assume here that skeletal muscle glycogen has been adequately super compensated (increasing intramyocellular water content and raising body weight) after a period of reduced carbohydrate intake that reduces body water content (and body weight) early in peak week (see above). If dehydration strategies result in a reduction scale weight that approximates or is below pre-carbohydrate loading levels, we hypothesize this reflects that the desired changes in the ECF (reduced subcutaneous fluid) and ICF spaces (increased intramyocellular fluid and glycogen) has been achieved.

**Table 4:** Example of Weight Change during Peak Week and Insights

	Body Weight (BW in lbs)		Note	Insight
Sunday (Start of Peak Week)		202	End of diet before peak week	
Wednesday AM (Pre-Glycogen Loading)		200	After 2–3 days of lower carbohydrate intake	
Friday AM (Post-Glycogen Loading)		205	After higher carbohydrate intake	Weight gain suggests glycogen super compensation
Saturday AM (Competition Day)	S C E N A R I O S	202	Clear urination and BW consistently falling	Further dehydration may improve appearance
		200	Minimal and darker urination with BW slowing	Water loss has been effective
		198	Urination ceased and BW constant	Very likely dehydrated, beware of being “flat”

**Fig. 2:**
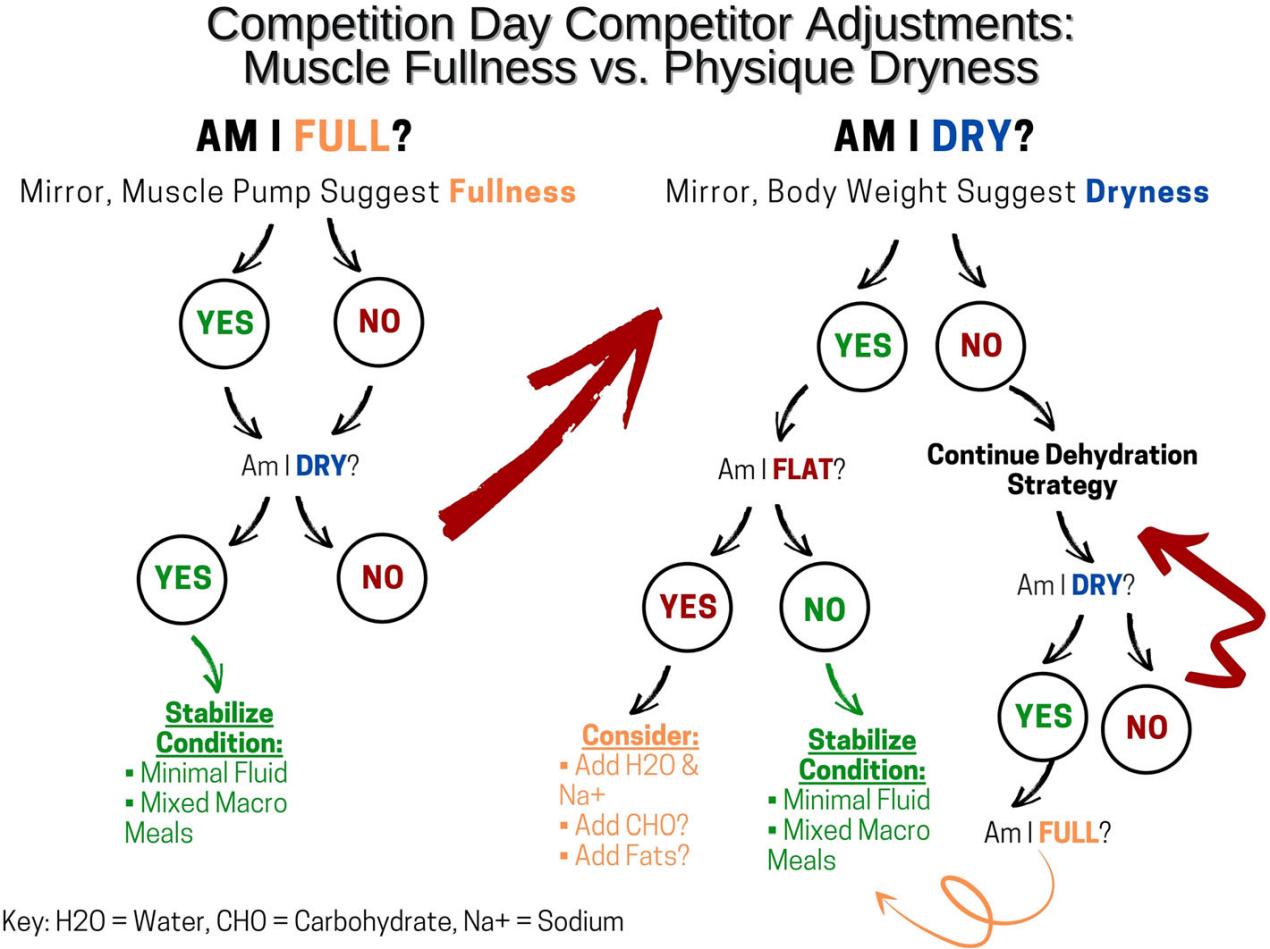
Decision Making Tree for Bodybuilding Competition Day Dietary and Fluid Adjustments

Figure 2 below outlines a competition day decision-making tree a competitor could employ to address the possibilities discussed above (lack of muscle fullness or physique dryness, or being “flat”). We presume a preference on minimizing body water over muscle fullness. Also, note that the scenario where “flatness” is an issue could require some combination of adding water, sodium, carbohydrate and/or dietary fat depending upon the circumstances. Previous mock peak week and carb-up experiences may serve the athlete well here in choosing an appropriate day of the show strategy. This same decision-making tree can be applied repeatedly in situations where the athlete is judged in multiple rounds.

## Conclusions

Evidence suggests that bodybuilders frequently use “peak week” strategies such as CHO loading, water/sodium manipulation, and other approaches in an attempt to enhance their physique during their last week of competition preparation. Unfortunately, there is a paucity of research on the effectiveness and safety of these strategies when implemented individually or collectively. Since the variables that are frequently manipulated by bodybuilders are interrelated, the alteration of one variable typically influences other variables. Furthermore, the inter-individual responses to the alteration of these variables makes it even more difficult to provide precise peak week “rules” to follow. Given the complicated interplay of physiological variables during peak week, as well as biological inter-individuality and variability in the importance placed on maximizing various aspects of muscularity across the different competitive divisions, there are a multitude of research avenues for investigating peak week strategies. In particular, tightly controlled examination of the quantifiable effects of glycogen supercompensation, graded dehydration via manipulation of sodium and/or water and pre-stage pump up strategies, coupled with documentation of the associated “practical,” subjective visual changes in physical appearance, would be relevant areas of study that may help better inform competitors and redirect them away from potentially dangerous and/or less effective peak week practices. Thus, the authors present this review and evidence-based approach to pre-contest peaking strategies based on the current state of the scientific literature in the hope it may spark further research, understanding and development of practical, safe approaches competitive bodybuilders can apply to optimize on-stage appearance.

## Data Availability

N/A.

## Abbreviations

ADH: Anti-diuretic hormone
PRN: As needed
ANF: Atrial natriuretic factor
HCO3^-^: Bicarbonate
Cl^-^: Chloride
CHO: Dietary carbohydrate
FAT: Dietary fat
PRO: Dietary protein
DHA: Docosahexaenoic acid
EPA: Eicosapentaenoic acid
ECG: Electrocardiogram
ECW: Extracellular water
FFM: Fat-free mass
FODMAPs: Fermented oligosaccharides, disaccharides, monosaccharides, and polyols
HDT: Head down tilt
IAAO: Indicator amino acid oxidation
ICF: Intracellular fluid
ICW: Intracellular water
IMT: Intramuscular triglycerides
JGA: Juxtaglomerular apparatus
MBIA: Multi-frequency bioelectrical impedance analysis
PO_4_^-^: Phosphate
K^+^: Potassium
RDA: Recommended dietary allowance
RAAS: Renin-angiotensin-aldosterone system
RE: Resistance exercise
Na^+^: Sodium
SGLTs: Sodium-glucose dependent cotransporters
TBO: Total body osmolarity
TBW: Total body water
